# A Q-Learning-Based Delay-Aware Routing Algorithm to Extend the Lifetime of Underwater Sensor Networks

**DOI:** 10.3390/s17071660

**Published:** 2017-07-19

**Authors:** Zhigang Jin, Yingying Ma, Yishan Su, Shuo Li, Xiaomei Fu

**Affiliations:** 1 School of Electrical and Information Engineering, Tianjin University, Tianjin 300072, China; zgjin@tju.edu.cn (Z.J.); myytju@tju.edu.cn (Y.M.); 2 School of Marine Science and Technology, Tianjin University, Tianjin 300072, China; fuxiaomei@tju.edu.cn

**Keywords:** underwater sensor networks, routing protocol, lifetime-extended, delay-aware, Q-learning technique

## Abstract

Underwater sensor networks (UWSNs) have become a hot research topic because of their various aquatic applications. As the underwater sensor nodes are powered by built-in batteries which are difficult to replace, extending the network lifetime is a most urgent need. Due to the low and variable transmission speed of sound, the design of reliable routing algorithms for UWSNs is challenging. In this paper, we propose a Q-learning based delay-aware routing (QDAR) algorithm to extend the lifetime of underwater sensor networks. In QDAR, a data collection phase is designed to adapt to the dynamic environment. With the application of the Q-learning technique, QDAR can determine a global optimal next hop rather than a greedy one. We define an action-utility function in which residual energy and propagation delay are both considered for adequate routing decisions. Thus, the QDAR algorithm can extend the network lifetime by uniformly distributing the residual energy and provide lower end-to-end delay. The simulation results show that our protocol can yield nearly the same network lifetime, and can reduce the end-to-end delay by 20–25% compared with a classic lifetime-extended routing protocol (QELAR).

## 1. Introduction

For decades, underwater wireless sensor networks (UWSNs) have attracted significant interest. Many applications of UWSNs, including commercial exploitation, marine mammal studies and oceanography data collection [1,2] allow humans to sense the vast underwater domain and motivate research on UWSN design.

However, because of the harsh environment and limited spectrum source, communications in UWSNs are much more difficult than those in terrestrial sensor networks. One of the reasons is that the radiowaves employed in terrestrial sensor networks is not feasible in the underwater environment because of their rapid attenuation. For example, Berkeley Mica 2 motes have been reported to have only 120 cm communication range in an underwater environment at 433 MHz [3]. Currently, the only appropriate method for long distance communications is acoustic communication. The speed of sound in water is about 1500 m/s, five-orders slower than the speed of radiowaves, thus there is a long propagation delay in UWSNs [4]. Moreover, the sensor nodes are deployed under the sea, therefore, it is difficult to recharge their batteries [5]. Since the sensors are powered by batteries, the limited energy restricts the network lifetime of UWSNs. Network lifetime is the time span from the deployment to the instant when the network is considered nonfunctional [6]. In this paper, the network lifetime is defined as the time span from the deployment to the instant the energy of the first node is exhausted. All these different characteristics make the algorithms, especially the routing algorithms used in terrestrial networks, unfeasible for UWSNs [7,8].

Typical routing algorithms employ shortest path algorithms for routing decisions. Thus, nodes chosen frequently on the shortest paths drain more quickly than other nodes, leading to a shorter network lifetime. To prolong the network lifetime, many routing algorithms are proposed. The energy efficient algorithm in [9] pays attention to coverage. It can preserve k-coverage and achieve maximal coverage for an area with the least energy consumption. However, low energy consumption does not necessarily lead to a long network lifetime. The distribution of residual energy also affects the network lifetime. In [10], the prolong stable election protocol (P-SEP) exploits the heterogeneity of energy thresholds to avoid low-energy nodes to nominate cluster heads and avoid continuous selection of a node. P-SEP has features like aliveness, fairness, full distribution in cluster head selection and can prolong network lifetime remarkably. However, like P-SEP, many routing algorithms employ greedy approaches to determine the next hop or the cluster head on the path, without considering the long-term rewards. That is to say, greedy routing algorithms only choose the node with the highest direct reward, even if the packet transmission thereafter needs more hops. Thus, the optimal next hop for the current node determined by these algorithms may not be the global optimal one for the whole routing path.

Moreover, the algorithms mentioned above pay no attention to the end-to-end delay, which is an important indicator in UWSNs. The fog-supported learning automata adaptive probabilistic search (FLAPS) algorithm in [11] is a delay-efficient distributed route-discovering algorithm. It can forward messages at the minimum bandwidth cost and latency. Although the synchronization functions in FLAPS are not suitable in UWSNs because of the long propagation delay in UWSNs, the application of Q-learning algorithm improves the performance remarkably. Nodes can process in an adaptive and distributed way using the reward-penalty mechanism of the Q-learning algorithm. With moderate improvement, the Q-learning algorithm can be implemented in UWSNs. The Min-delay routing in [12] based on the Dijkstra algorithm can minimize delay, reduce link interruption and improve reliability. However, it is a multipath routing method, which means that there is more than one path from the source node to the sink node. Thus, Min-delay routing may increase the energy consumption. Above all, in order to prolong the network lifetime, nodes with more residual energy should be chosen as relay nodes even though they are far from the sink node, while in order to minimize the end-to-end delay, nodes near to the sink node should be chosen. Network life and end-to-end delay are both important in UWSNs. Therefore, it is necessary to introduce significant compromises at the routing design stage.

To cater for these issues, we propose a Q-learning-based delay-aware routing (QDAR) mechanism to extend the network lifetime for UWSNs. In QDAR, nodes only need to know about their residual energy and the delay from neighbors. As the action-utility function (Q-value) of Q-learning technique takes both direct reward and discounted long-term reward into account, Q-learning-based protocols can determine the global optimal next hop instead of a greedy one. The main contributions of QDAR can be summarized as follows: (1) it defines a data collection phase and designs the packet structure before routing decisions to quickly adapt to the dynamic underwater environment; (2) it takes both delay and residual energy into consideration by defining two kinds of cost functions: delay-related cost and energy-related cost; (3) it uses an adaptive mechanism to ensure a longer network lifetime and a relatively shorter delays: when the residual energy is enough, the end-to-end delay is restricted, while the residual energy of some nodes is lower than the threshold, an adequate path consisting of nodes with longer delays but more remaining energy is determined; (4) QDAR is easily extendible: energy consumption, channel capacity, communication reliability and many other metrics can be integrated into the action-utility functions in future research for different targets.

The QDAR algorithm can work adaptively and distributively through trade-offs between the network lifetime and end-to-end delays. The simulation results show that our algorithm achieves nearly the same network lifetime extension as the existing lifetime-extended protocol, and reduces end-to-end delay by 20–25%.

The rest of this paper is organized as follows: in Section 2, related works on underwater routing protocols are discussed briefly. In Section 3, the basic Q-learning technique is introduced and adopted into our system model. In Section 4 and Section 5, the QDAR algorithm is described in detail. The simulation results are shown and discussed in Section 6. Finally, we conclude this paper in Section 7.

## 2. Related Work 

Underwater routing techniques are a hot research topic for UWSNs nowadays. There are several kinds of routing protocols that aim to improve energy efficiency, reduce end-to-end delay and prolong network lifetime [13,14]. In this section, we provide a review on research works that have been done on this topic.

Most energy-efficient routing protocols aim to reduce energy consumption and prolong network lifetime. A hierarchical routing algorithm called queen-bee evolution algorithm (QEGA) [15] works better in terms of energy consumption. QEGA has a high rate which results in premature convergence. Thus, the algorithm can find the optimal solution more quickly. However, QEGA does not consider the residual energy, which is important to extend network lifetime. The energy-saving vector-based forwarding (ES-VBF) protocol [16] defines a desirableness factor based on residual energy and location information. In the routing pipe, nodes with more residual energy are more possible to forward packets. Although the algorithm prolongs the network lifetime, it needs the location information of all the nodes, which is still a challenge to be solved.

The adaptive power controlled routing (APCR) [17] is an energy efficient routing schema that does not require any location information. In APCR, nodes are assigned to concentric layers according to the signal power of a received INTEREST packet broadcasted by sink nodes. Then, routing paths are decided based on layer numbers and residual energy. To improve the energy efficiency, nodes are able to adjust their transmission power to a set of values according to the information received during packet transmission. If forwarding nodes are found at multiple layers, the power is decreased. If no neighbor is found, the power is increased. Thus, APCR can achieve a high delivery ratio, but the number of forwarding nodes at each layer is not limited properly. If multiple nodes forward the same packet, the total energy consumption is increased.

The Q-learning-based adaptive routing (QELAR) protocol is proposed [18] and Q-learning is proved to perform well in UWSNs in several aspects. QELAR defines the reward function based on the residual energy of the sensor nodes. In this protocol, sensor nodes choose the node with more residual energy as the next hop, so that the network lifetime of the network can be extended. However, in QELAR protocol, each node takes the responsibility to learn the environment by metadata exchanging and decide the next hop, leading to a higher energy consumption for each node. Moreover, the protocol does not restrict end-to-end delay. When the number of the sensor nodes increases, the routing will detour with more and more nodes, then the end-to-end delay is prolonged. Thus, QELAR works inefficiently in some situations because of the long delay.

Many research works point out that the problems of latency in UWSNs are serious, especially for time-critical applications. In [19] the authors employ a probability model to describe the propagation delay of a link and select the next hop with lower delay. In [20], an underwater opportunistic routing (UWOR) is proposed. The forwarding set in which nodes can hear each other and prevent packet duplication is established. Each node in the forwarding set is assigned a relay priority which is related to the probability of successful transmission. The node with the highest priority and limited end-to-end delay can be chosen as the relay node. The simulation results show that UWOR can maximize good put while satisfying end-to-end latency requirements. However, it disables retransmission mechanisms, leading to a lower delivery ratio.

Moreover, there are protocols that can jointly reduce energy consumption and end-to-end delay. Modified energy weight routing (MEWR) protocol [3] is energy efficiency guaranteed using a minimum algorithm. In order to determine an optimal path with a low end-to-end delay as well as low energy consumption, the cost of a link is formulated as a mixture of energy weight and delay weight. In the path discovery phase, a node employs a greedy approach to find all its neighbors and determine an optimal one with the lowest cost. However, low energy consumption does not lead to long network lifetime effectively. Since MEWR does not take the residual energy of sensor nodes into account, it cannot optimize the energy distribution, which is crucial for network lifetime extension.

## 3. Q-learning Based Model

In this section, we give a brief introduction to the basic Q-learning technique, which is the fundamental theory of our algorithm. Next, we explain our Q-learning based system model.

### 3.1. The Basic Q-Learning Technique

Q-learning is a model-free reinforcement learning method. It provides agents with the capability of learning to act optimally. In this technique, an agent chooses an action at a particular state according to the reinforcement it receives. The reinforcement is composed of the direct reward or penalty and the future consequence the agent estimates. With the reinforcement, the agent can evaluate how good an action is in the current situation. The task of the Q-learning is to determine an optimal policy to get a higher reward. Once the agent receives the highest reward, it will stop at the state which is called absorbing goal. An agent in state *x_n_* at step *n* can go to the next state *x_n+1_* by choosing action *a_n_* under the policy π. The probability of shifting to state *x_n+1_* according to the law:

(1) $$P_{x_{n}x_{n+1}}^{a_{n}}=prob\{x=x_{n+1}|x_{n},a_{n}\}$$

The agent receives rewards according both the values of direct reward and the future reward. A direct reward *r_n_* in state *x_n_* is received immediately after the agent performs action *a_n_*, only depending upon the state and action. A future reward is the total reward that the agent expects in the new state after action *a_n_*. The action-utility function $Q^{\pi }\left(x,a\right)$, which is used to describe the expected return of action *a* in state *x* under policy *π*, is critical to Q-learning:

(2) $$Q^{\pi }\left(x_{n},a_{n}\right)=r_{n}+\gamma \sum_{x_{n+1}\in X}P_{x_{n}x_{n+1}}^{a_{n}}Q^{\pi }\left(x_{n+1},a\right)$$

where:

(3) $$r_{n}=\sum_{x_{n+1}\in X}P_{x_{n}x_{n+1}}^{a_{n}}R_{x_{n}x_{n+1}}^{a_{n}}$$

$R_{x_{n}x_{n+1}}^{a_{n}}$ is the reward of action *a_n_*, which can make the agent go into state $x_{n+1}$ from state $x_{n}$. γ(0≤γ<1) is the discount factor. It means the reward received current step hence is worth less than that received now. Typically, to balance the direct and future reward, the value of γ is within (0.5, 0.99). If an optimal policy is performed thereafter, we can derive the optimal Q value. It is proved that at least one optimal stationary policy $\pi^{*}$ exists [21]. Under the optimal policy, $Q^{*}$ can be described as:

(4) $$Q^{*}\left(x_{n}\right)={max}_{a}\left(r_{n}+\gamma \sum_{x_{n+1}\in X}P_{x_{n}x_{n+1}}^{a_{n}}Q^{*}\left(x_{n+1},a\right)\right)$$

Thus, in order to get an optimal *Q*, we have:

(5) $$Q\left(x_{n},a_{n}\right)=r_{n}+\gamma \sum_{x_{n+1}\in X}P_{x_{n}x_{n+1}}^{a_{n}}Q^{*}\left(x_{n+1}\right)$$

and:

(6) $$a_{n}=\arg\;\max Q\left(x_{n},a_{n}\right)$$

where *a_n_* is the optimal action to get the optimal *Q*.

### 3.2. Q-Learning Based System Model

In the UWSNs, the sink nodes are on the surface while the source nodes are deployed underwater. The sink nodes can receive data from multiple distributed source nodes [22]. In our QDAR mechanism, when a source node has a packet to send to the sink node, it broadcasts to the sink node to request communication as well as collect information. This mechanism is explained in the next section. With the collected information, the sink node can lay out a virtual topology from the source node to the sink node and decide the routing path thereafter.

Each packet in the networks can be seen as an agent in the Q-learning technique. The Q-learning state is related to the node which holds the packet. When a packet is at node *i*, the state of the agent is *x_i_*. Packet forwarding from node *i* to node *j* is action *a_i_*. If the transmission is successful, the packet (agent) state shifts from *x_i_* to *x_j_*. The routing path is the policy π, directing the packet (agent) to take the proper action. Obviously, it is costly for a node to perform all actions by sending packets to all the neighbor nodes and get all these *Q* values. Therefore, in our mechanism, the sink assumes a virtual packet and sends it in the virtual topology. By doing this virtual experiment, the sink node can perform QDAR algorithm and determine the routing path because the sink holds the nodes information, such as the delay and the residual energy.

## 4. QDAR Mechanism Overview

### 4.1. The QDAR Mechanism

**Figure 1:**
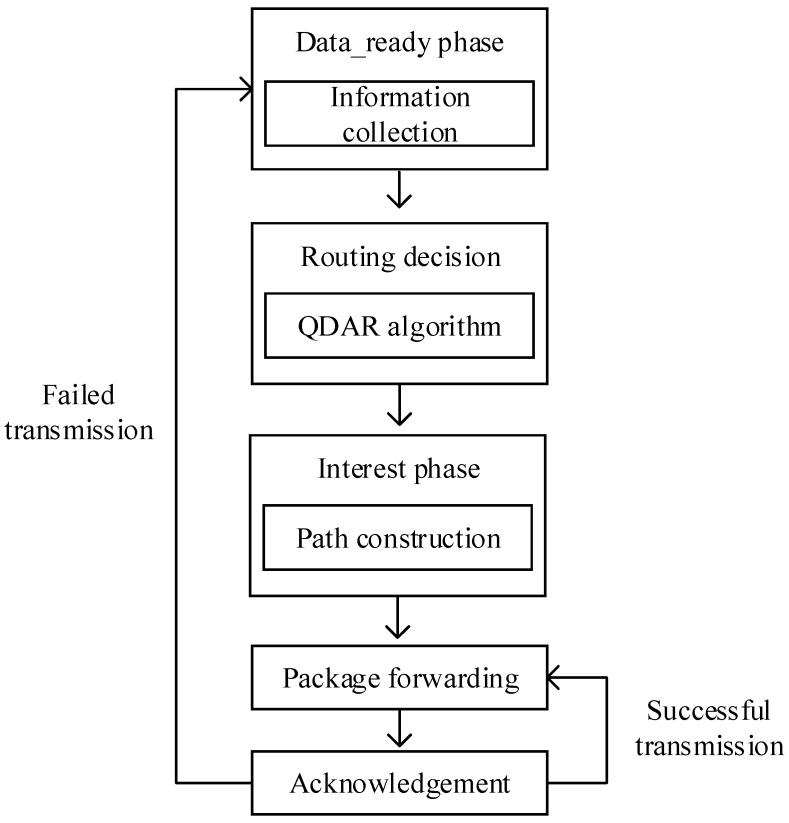
Q-learning based delay-aware routing mechanism.

The QDAR mechanism is designed for the overall routing process. There are five phases in QDAR mechanism: data_ready phase, routing decision, interest phase, packet forwarding and acknowledgement. The data_ready phase is responsible for information collection, after which the sink node can get the fundamental information for routing decision. Then, the sink node determines the path according to QDAR algorithm. During the interest phase, the sink node sends a packet to the source node along the determined path. Thus, the path can be constructed. Packets are sent along the path thereafter as long as the acknowledgement phase is successful. Otherwise, the whole mechanism restarts. The main communication procedure is briefly depicted as Figure 1.

### 4.2. Assumptions 

The following assumptions apply:

1. Nodes hold their own depth information and can embed it in the packets;
2. Nodes in UWSNs implement Source_initiated Query [23];
3. The sink node keeps the successful and failed communication record of the nodes.

In the Source_initiated Query, if a source node has a packet to send to the sink, it first broadcasts a DATA_READY packet for both communication request and information collection. This phase is defined as the data_ready phase in this paper. During the data_ready phase, a node only forwards the packet if its depth is smaller than that of the previous node.

Once the sink node receives this packet, it can decide if it is interested in such data. If so, the sink node decides the routing path according to the QDAR algorithm and sends out an INTEREST packet to the source node along the decided path for path construction. This phase is defined as the interest phase. In this phase, each node records its previous hop and takes it as the next hop in the packet forwarding phase. Only nodes selected by QDAR algorithm join in the INTEREST packet forwarding, while the others turn into sleep state thereafter or take responsibilities for another communication task to save energy or improve network capacity.

After the INTEREST packet reaches the source node, the path is constructed. Then, the source node transmits the data packet through the constructed path. Finally, the sink node responds acknowledgement (ACK) packet after the successful data reception. If the source node receives ACK successfully, this communication episode concludes. The coming packets from the same source node are sent along the same path without repeated data collection and routing decision phases until transmission failure occurs.

### 4.3. The Packet Structures

Packet structures for DATA_READY packet and INTEREST packet, which can help establish the routing track, are designed as follows. DATA_READY packet contains the type and ID of the packet, source node address (SNA), time stamp (TS), current node depth (CND) and two arrays C and RES in which two costs and residual energy of this node (RE) are saved respectively. SNA is permanent during the network lifetime of the packet. Elements of C are calculation results of two cost functions: delay-related cost and energy-related cost. Denoting the residual energy of node *i* as $e_{res}^{i}$, we define the energy-related cost function $ce\left(e_{res}^{i}\right)$ as:

(7) $$ce\left(e_{res}^{i}\right)=1-\frac{e_{res}^{i}}{e_{ini}^{i}}$$

where $e_{ini}^{i}$ is the initial energy of node *i*. The less energy a node remains, the more it costs to forward packets. A node with higher cost is more reluctant to communicate. A delay-related cost function $ct\left(t_{ij}\right)$ is also defined:

(8) $$ct\left(t_{ij}\right)=1-\frac{1}{t_{ij}+1}$$

where $t_{ij}$ represents the delay of sending packet between node *i* and node *j*. Clearly, the longer delay, the higher cost. On receiving a DATA_READY packet, the nodes have two tasks:

1. Calculate. The nodes extract RE and TS. With these information, the nodes can compute their energy-related costs and delay-related costs depending on the delay and residual energy.
2. Update and relay. The nodes update TS, C and RES packet fields with their own information or calculation results and relay the DATA_READY packet to neighbor nodes until the packet arrives at the sink node.

**Figure 2:**
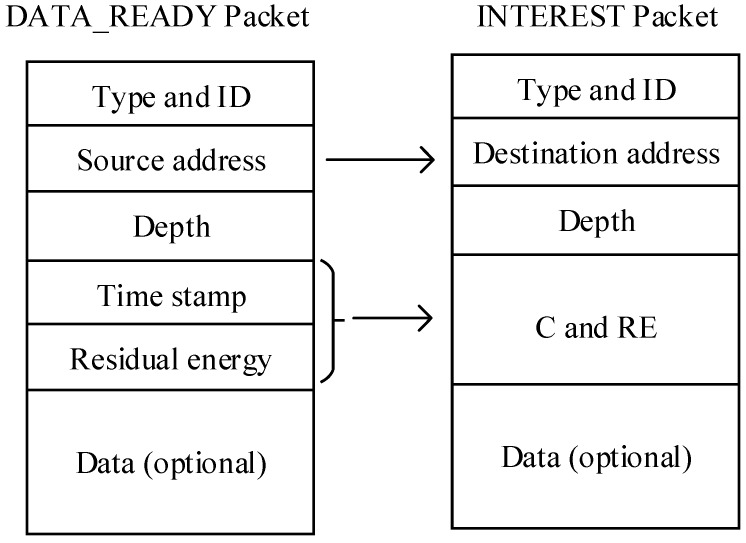
Packet structures of DATA_READY packet in data_ready phase and INTEREST packet in interest phase and their relationship.

When the DATA_READY packet reaches the sink node, the sink node holds the relevant data and begins routing decision if it is interested in the packet. After the routing decision phase, the sink node creates an INTEREST packet composed of type and packet ID, destination node address (DNA), current node depth (CND) and path direction (PD). DNA is the source node address (SNA) of DATA_READY packet. The structures of both packets and their relationship are shown in Figure 2.

## 5. QDAR Algorithm

In this section, we describe the proposed QDAR algorithm in details. The important notations are listed in Table 1.

In order to design a Q-learning-based delay-aware routing protocol to extend the network lifetime of UWSNs, we define an action-utility function whose value is Q. The sink node has a matrix in which Q values of all the nodes are stored. These Q values are future rewards of packet forwarding and are used in the routing decisions. In our protocol, node *i* and packet forwarding from node *i* to node *j* are seen as state *i* and action *j* of Q-learning technique, respectively.

Firstly, we define a reward function $R_{s_{ij}}^{a_{j}}$ for action *j*, which is related to both propagation delay and residual energy. If this transmission is successful, the reward of the action for node *i* is:

(9) $$R_{x_{ij}}^{a_{j}}=-\beta_{0}-\beta_{1}C_{ij}=-\beta_{0}-\beta_{1}\left[ce\left(e_{res}^{i}\right)+{\varphi_{ij}}^{*}\;ct\left(t_{ij}\right)+ce\left(e_{res}^{j}\right)\right]$$

where $a_{j}$ is action *j*. Because forwarding packet occupies channel bandwidth and disturbs other nodes, a constant cost $\beta_{0}$ is added into the function. $\beta_{1}$ is the weight of the sum of delay-related cost and energy-related cost. $\varphi_{ij}$ is the delay sensitivity. A higher $\varphi_{ij}$ means the delay is more repellent.

If the transmission fails, node *i* will resend the packet, which means the node should pay double energy-related cost and more delay-related cost. Thus, the reward function becomes:

(10) $$R_{x_{ii}}^{a_{j}}=-\beta_{0}-\beta_{2}C_{ii}=-\beta_{0}-\beta_{2}\left[2\times ce\left(e_{res}^{i}\right)+{\varphi_{ij}}^{*}\;ct\left(t_{ij}^{\prime }+t_{ij}\right)+ce\left(e_{res}^{j}\right)\right]$$

where $t_{ij}^{\prime }$ is the time that node *i* spends in the failed transmission. $\beta_{2}$ is the weight of the sum of delay-related cost and energy-related cost, in the same position as $\beta_{1}$.

To further prolong the network lifetime, we design an adaptive detouring path strategy. We define a set $N_{i}$ for node *i* and an energy warning threshold $e_{\mathrm{wth}}$. The elements of $N_{i}$ are neighbor nodes of node *i*. When the residual energy of the next hop of node *i*, for example, node *j*, is lower than $e_{\mathrm{wth}}$, or the energy-related cost $ce\left(e_{res}^{j}\right)>ce\left(e_{\mathrm{wth}}\right)$, the sink node modifies the $\varphi_{ih}{|}_{h\in N,\;h\neq j}$ values to $\varphi_{ih}^{\prime }{|}_{h\in N,\;h\neq j}$, where $\varphi_{ih}^{\prime }{|}_{h\in N,\;h\neq j}=\varphi_{0}\varphi_{ih}{|}_{h\in N,\;h\neq j}$, $0<\varphi_{0}<1$. In this way, the weight of delay-related cost of communication with node *j* is higher than those of the other nodes. With $\varphi_{ih}^{\prime }$, the sink node can determine a detouring path by choosing nodes with more residual energy as the next hop of node *i*.

To calculate the direct reward, sink node keeps the communication record so as to estimate the state transition probabilities: $P_{x_{ij}}^{a_{j}}$ and $P_{x_{ii}}^{a_{j}}$ of each node. $P_{x_{ij}}^{a_{j}}$ and $P_{x_{ii}}^{a_{j}}$ are the probabilities of successful and failed packet forwarding respectively:

(11) $$P_{x_{ij}}^{a_{j}}=\frac{m_{t}}{M_{t}},$$

(12) $$P_{x_{ii}}^{a_{j}}=1-P_{x_{ij}}^{a_{j}}.$$

Suppose there have been $M_{t}$ instances of communication up to time t and $m_{t}$ is the frequency of successful packet forwarding, we can define the direct reward function as:

(13) $$r_{i}=R_{x_{ij}}^{a_{j}}P_{x_{ij}}^{a_{j}}+R_{x_{ii}}^{a_{j}}P_{x_{ii}}^{a_{j}}=R_{x_{ij}}^{a_{j}}\frac{m_{t}}{M_{t}}+R_{x_{ii}}^{a_{j}}\left(1-\frac{m_{t}}{M_{t}}\right)$$

The highest Q value among all the actions is described as:

(14) $$Q^{*}\left(x\right)=\max_{a}Q\left(x,a\right)$$

If the next hop is the sink node, the Q of this node is much higher than the others. According to Equations (5) and (14), we can define the action-utility function for each neighbor node of node *i* as:

(15) $$Q\left(x_{i},a_{j}\right)=r+\gamma \left(\frac{m_{t}}{M_{t}}Q^{*}\left(x_{j}\right)+\left(1-\frac{m_{t}}{M_{t}}\right)Q^{*}\left(x_{i}\right)\right)$$

Then, we choose the neighbor node with the highest Q as the next hop and update the previous Q storied with newly chosen $Q^{*}$. Initially, if the next hop is not the destination node, *Q* values in the matrix are set to 0. Otherwise, Q values are set to 1. Algorithm 1 for the routing mechanism is conducted as below.
**Algorithm 1:** The routing mechanism.  Initialize Q();  **While**
*x_i_*. next_hop ! = source node    **for**
*x_j_* in *N_i_*
**do**     calculate *ce*, *ct*, *P*;    nodes satisfy $ce\left(e_{\mathrm{wth}}\right)<ce\left(e_{res}^{j}\right)$ are saved in set $N_{i0}$;  **end for**  **if**
$N_{i0}!\;=\;\emptyset$
**then**  set $\varphi_{ih}$ to $\varphi_{ih}^{\prime }$, $h\in N_{i}/\left(N_{i0}\right)$;  calculate the direct reward *r*;  select the node *x_j_* with maximum Q value in set *N_i_*;  calculate Q(*x_i_, a_h_*),$\;h\in N_{i}/\left(N_{i0}\right)$;  *a_j_* = *argmax*(Q(*x_i_*, *a_h_*));  **else**
*a_j_* = *argmax*(Q(*x_i_*, *a_h_*))  **end if**  *x_i_* = *x_j_*

The QDAR mechanism can adapt to the dynamic underwater environment quickly. An energy-related cost function and a delay-related cost function are defined in the data_ready phase. After that, the sink node has the collected information and performs QDAR algorithm. Firstly, it defines two reward functions with the cost functions for both successful and failed transmission. In the reward functions, there is an alterable parameter. Based on this parameter, an adaptive detouring path strategy is designed. Thus, QDAR algorithm can work adaptively with different residual energy. Then, with the reward functions and the corresponding probability functions, the action-utility function (Q-value) is determined.

Finally, the sink node chooses the global optimal next hop and then determines the routing path. In the next section, the simulation results prove that the adaptive solution in QDAR ensures a longer network lifetime and a relatively shorter delay.

## 6. Performance Evaluation

In this section, we evaluate the performance of the QDAR algorithm. Firstly, the performance of the QDAR algorithm with different parameters is shown. Then, we compare QDAR with the Q-learning-based lifetime-aware (QELAR) routing protocol and the vector-based forwarding (VBF) routing protocol. Finally, the performances of QDAR and QELAR are compared with different underlying MAC protocols.

### 6.1. Experimental Framework

In our simulation, 80 sensor nodes are randomly deployed in a 5000 m × 5000 m × 1500 m three-dimensional space. These sensor nodes are identical in every feature, such as the initial energy, communication power, transmission range and so on. Five sink nodes are randomly deployed on the water surface. As it is convenient to maintain these sink nodes, we assume that these sink nodes have infinite energy and can communicate with each other by radio. Thus, packets can be sent to any sink node. We place a source node at the bottom layer of the network. The transmission speed is 1500 m/s. At the source node, packets are generated and prepared to send to the sink node following an independent Poisson process with a rate λ (packets/s). The initial energy, energy warning threshold are set to 150 J and 75 J. Because $\beta_{1}$ and $\beta_{2}$ are in equal positions, they are set to the same value: $\beta_{1}=\beta_{2}$. The constant cost $\beta_{0}=1$ and $\varphi_{ij}=1$. We use $\varphi_{0}$ to restrict the detour routing. The underlying MAC protocol is the underwater power control (UPC) protocol [24]. Moreover, the preamble signal length should be considered in the end-to-end delay as it is long in UWSNs. For example, the preamble of the Aqua-sent OFDM is 0.49 s, significantly increasing the end-to-end delay. In accordance with the hardware specifications of underwater OFDM modems [25], the simulation parameters are listed in Table 2.

### 6.2. Evaluation with Different Parameters

To evaluate the influence of $\varphi_{0}$, $\beta_{1}$ and $\beta_{2}$, we simulate the variance of residual energy and the average latency of each packet as these values change. $\beta_{1}$ and $\beta_{2}$ are the weights of the sum of delay-related cost and energy-related cost for successful and failed transmission respectively. $\varphi_{ij}$ is the delay sensitivity. $\varphi_{0}$ is the coefficient to modify $\varphi_{ij}$. Higher $\beta_{1}$ and $\beta_{2}$ can distribute energy more evenly because the residual energy is more important in routing decision. Higher $\varphi_{0}$ means delay is more important so that the detouring routing is restricted.

**Figure 3:**
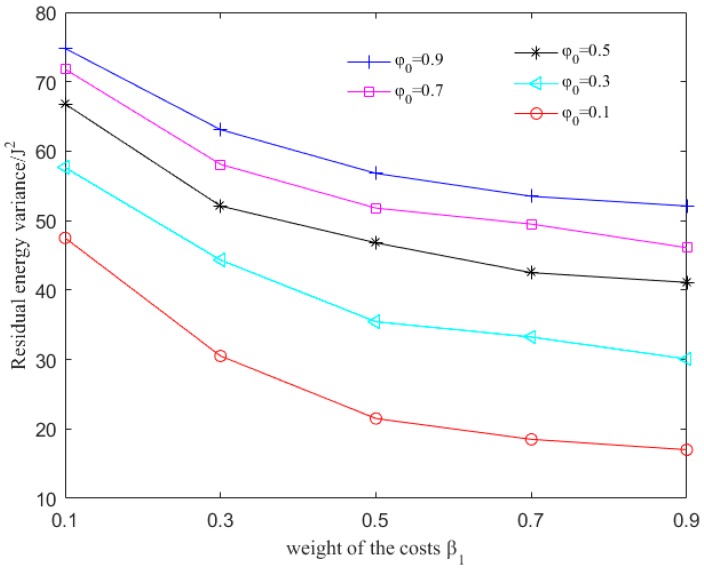
Variance of the residual energy with different values of $\varphi_{0}$, $\beta_{1}$ and $\beta_{2}$.

In the simulation, $\varphi_{0}$, $\beta_{1}$ and $\beta_{2}$ vary between 0.1 and 0.9 with a step of 0.2. Comparing the variance of residual energy in Figure 3 with varying parameters, we can conclude that with larger $\beta_{1}$, $\beta_{2}$ and smaller $\varphi_{0}$, the residual energy distributes more uniformly. When $\beta_{1}=\beta_{2}=0.9$ and $\varphi_{0}=0.1$, the residual energy variance is about 18, less than 1/3 of that with $\beta_{1}=\beta_{2}=0.1$ and $\varphi_{0}=0.9$. That is to say less nodes will drain the energy early. Thus, the network lifetime can be prolonged.

**Figure 4:**
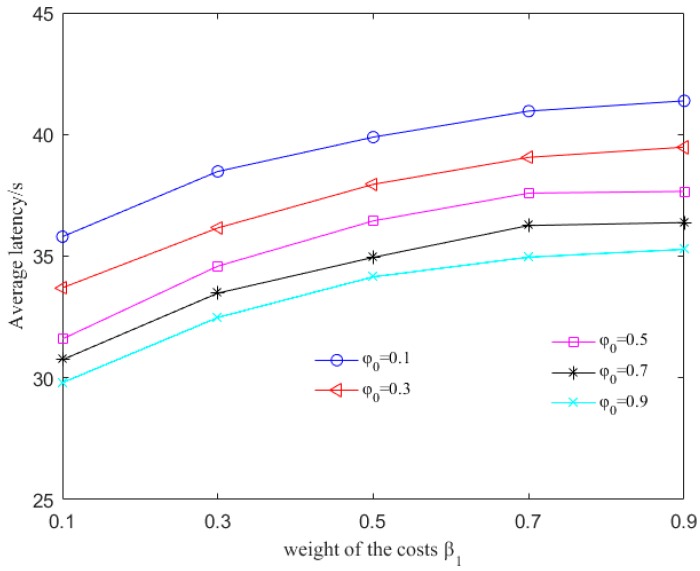
Average latency with different values of $\varphi_{0}$, $\beta_{1}$ and $\beta_{2}$.

However, as shown in Figure 4, the end-to-end delay is increased. When $\beta_{1}=\beta_{2}=0.9,\;\varphi_{0}=0.1$, the delay is 42 s, about 25% longer than that with $\beta_{1}=\beta_{2}=0.1,\;\varphi_{0}=0.9$. This is because that the path detours to avoid nodes close to the sink node but with relatively lower energy. On one hand, higher $\beta_{1}$ and $\beta_{2}$ mean higher weight of the energy-related cost. Nodes with more residual energy cost less to communicate. Therefore, they are more favorable to packet forwarding, making the variance of residual energy lower. On the other hand, as $0<\varphi_{0}<1$, $\varphi_{0}$ indicates that the sink node attaches less importance to delay during the routing decision. Although a node is far away from the sink node, it can be the next hop as long as the residual energy is high enough. As a result, detour routing with more residual energy and longer latency is chosen as the routing path. Furthermore, as $\varphi_{0}$ increases, the rates of the incremental variance and decreasing average latency reduce. The reason is that, when $\varphi_{0}$ increases to a certain degree, the sink node determines routing path with the lowest latency thereafter, paying little attention to the residual energy. However, when $\varphi_{0}$ is too small, the average latency increases significantly because of the detour routing. Thus, $\varphi_{0}$ can not increase or decrease infinitely. In the following evaluation, we set $\varphi_{0}$ to 0.3, 0.5 and 0.7 respectively and set $\beta_{1}$, $\beta_{2}$ to 0.7.

### 6.3. Comparison with QELAR and VBF

**Figure 5:**
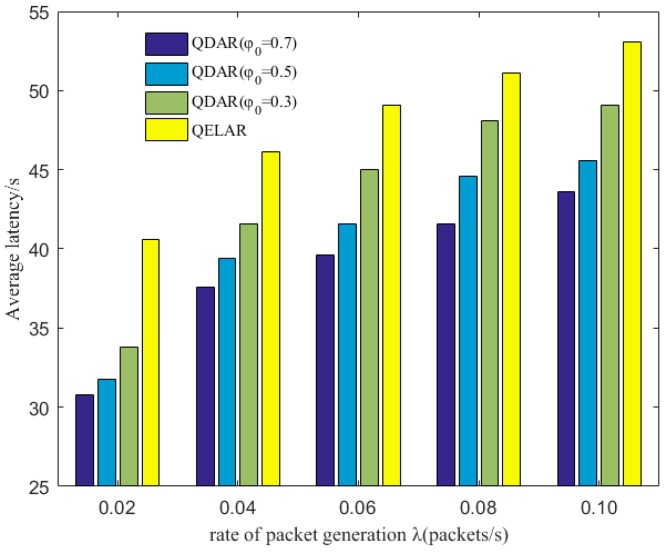
Average latency comparison between QDAR and QELAR with different packet generation rate λ (packets/s).

Figure 5 depicts the average latency of each package with different $\varphi_{0}$ and the comparison with the QELAR protocol, a Q-learning-based lifetime-extended protocol. As λ increases, more packets need to be forwarded and the average latency is prolonged. For example, when $\varphi_{0}=0.3$, the latency increases about 23% with λ grows from 0.02 to 0.1. This is because both QDAR and QELAR choose detour routing to distribute the residual energy evenly. When there are more packets sent in UWSNs, nodes closer to the sink node have relatively less residual energy. Thus, the nodes far away from the sink node but with more residual energy are chosen to relay packets.

It is also observed that, in UWSNs with a lower λ, QDAR can reduce latency more significantly. Especially when $\varphi_{0}=0.7$, the latency is reduced by nearly 25%. The reason is that at the initial stage of communication, although the residual energy reduces, it is still sufficient and does not worth detour routing. However, QELAR detours from the outset at the initial stage, while QDAR waits until the residual energy of some nodes is lower than a threshold. The higher the $\varphi_{0}$ is, the more attention is paid to latency. Another reason is that, in QDAR, it is the sink node that takes responsibility to construct routing path. Whereas in QELAR, every node in the network should learn the environment and choose an optimal action, leading to a longer delay. In this way, QDAR outperforms QELAR in the average latency by 20–25% as shown in Figure 5.

**Figure 6:**
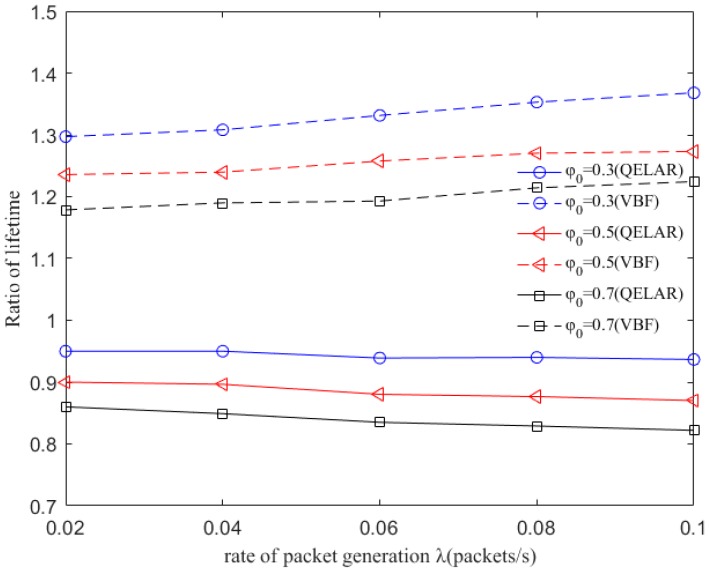
Lifetime ratios of QDAR and different routing protocols (QELAR and VBF) with different packet generation rate λ (packets/s).

Since the network lifetime is another critical performance of network, we compare the network lifetime of QDAR, QELAR and VBF protocol [23]. The network lifetime is defined as the total performing time until the first node drains its energy. The ratio of QDAR network lifetime and QELAR network lifetime as well as the ratio of QDAR network lifetime and VBF network lifetime is shown in Figure 6. As depicted, the ratio of QDAR network lifetime and VBF network lifetime is about 1.2 with $\varphi_{0}=0.7$. When $\varphi_{0}=0.3$, the ratio increases to 1.3. This is because in VBF, all the nodes in the virtual pipe between a source and a destination have chances to relay packets. As a result, many packets are sent repeatedly by different nodes, making them drain energy more quickly. With λ growing from 0.02 to 0.1, more packets are sent and the nodes within the pipe die earlier. On the contrary, QDAR takes the residual energy into account and extends the network lifetime by avoiding to choose nodes with relative less energy. The ratio of QDAR network lifetime and QELAR network lifetime is smaller than 1. The reason is that QELAR can distribute routing traffic to nearly every node in UWSNs, extending the lifetime greatly. While QDAE also take delay into account, avoiding choosing nodes that are too far away from the sink node to forward packets. Thus, some nodes may exhaust energy earlier than that in QELAR. However, even for the worst case, the network lifetime can still achieve 82% of that in QELAR. The factor attributes to the result is our adaptive detouring path strategy: if the residual energy of a node is less than $e_{\mathrm{wth}}$, an adequate path with nodes remaining more energy is chosen to relay packets, to postpone energy depletion of the node.

### 6.4. Evaluation with Different MAC Protocols

**Figure 7:**
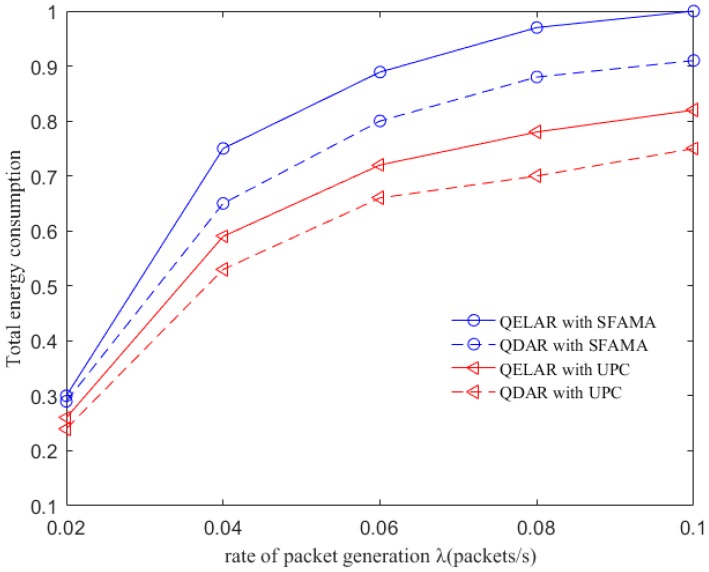
Total energy consumption between QDAR and QELAR with different MAC protocols.

We next examine how the underlying MAC protocol layer affects the performance of QDAR and QELAR in terms of total energy consumption. The slotted floor acquisition multiple access (SFAMA) MAC protocol [26] and underwater power control (UPC) MAC protocol [24] are used for QDAR and QELAR, respectively. The results are shown in Figure 7, where the total energy consumption is normalized to (0, 1). As QDAR restricts routing decision with end-to-end delay, some packets may detour with more hops in QELAR than that of QDAR. More nodes forwarding packets means more total energy consumption. Thus, QELAR consumes more energy than QDAR regardless of the MAC protocol used. Besides, we can see that routing algorithms with UPC can achieve lower energy consumption than those with SFAMA. The reason is that UPC can reduce the transmission power level to achieve a better energy efficiency as well as smaller interference range. Moreover, as UPC can deal with the noise, interference and spatial channel reuse, QDAR do not need to pay much attention to these problems. Therefore, using UPC as the underlying MAC protocol, QDAR can prolong the network lifetime more efficiently.

## 7. Conclusions

In this paper, we have designed a novel delay-aware routing (QDAR) mechanism based on the Q-learning technique to extend the network lifetime of underwater sensor networks. The QDAR mechanism can reduce average latency as well as extend the network lifetime. The data_ready phase and the packet structures are designed for data collection. Then, the sink node applies the QDAR algorithm to determine the routing path. In QDAR, we extend the action-utility function with both residual energy-related cost and delay-related cost. In order to have a better tradeoff between residual energy of nodes and delay, an adaptive detouring path strategy is designed. When the residual energy is sufficient, a path with shorter delay is chosen. When the residual energy of a node is lower than a threshold, the weight of the delay-related cost is decreased so as to construct an adequate path avoiding nodes with relatively less energy, even though these nodes may be nearer to the sink node. Thus, QDAR can distribute the residual energy more evenly, which is crucial to extend the network lifetime. Moreover, as QDAR takes both direct rewards and future rewards into account, it can choose a global optimal next hop, whereas greedy algorithms only pay attention to the direct reward. After a routing decision, the path is constructed during the interest phase. Then, packets are forwarded and the communication ends with an acknowledgement. The QDAR mechanism can work adaptively and distributively in the dynamic underwater environment. We evaluate the performance of QDAR with different parameters and compare it with QELAR and VBF. The simulation results show that QDAR reduces the total energy consumption effectively and decreases the average latency significantly by 20–25% at the cost of only a little reduction in network lifetime. Therefore, QDAR is more adequate for time-critical applications.

## Figures and Tables

**Table 1:** Notations.

Name	Description
$a_{j}$	Packet forwarding from node *i* to node *j*
$\beta_{0}$	Constant cost
$\beta_{1}$, $\;\beta_{2}$	Weight of two costs
$N_{i}$	The neighbor node set of *i*
$\varphi_{ij}$	Delay sensitivity of communication between node *i* and *j*
$\varphi_{ij}^{\prime }$	The modified $\varphi_{ij}$
$t_{ij}$	Delay of communication between node *i* and *j*
$t_{ij}^{\prime }$	The time of failed communication between node *i* and *j*
$ce\left(e_{res}^{i}\right)$	The energy-related cost of node *i*
$ct\left(t_{ij}\right)$	The delay-related cost of node *i*
γ	The discount factor of future reward

**Table 2:** Simulation parameters.

Name	Values
Transmission power	10 W
Receiving power	3 W
Idle power	30 mW
Data packet size	300 B
Transmission rate	3 kbps
Transmission range	500 m
Preamble signal length	0.49 s
Simulation time	10^4^ s

## References

[1] Felemban E., Shaikh F.K., Qureshi U.M., Sheikh A.A., Qaisar S.B. (2015). Underwater sensor network applications: A comprehensive survey. Int. J. Distrib. Sens. Netw..

[2] Sheikh A.A., Felemban E., Felemban M., Qaisar S.B. Challenges and opportunities for underwater sensor networks. Proceedings of the 12th IEEE International Conference on Innovations in Information Technology (IIT).

[3] Zhang S., Wang Z., Liu M., Qiu M. (2014). Energy-aware routing for delay-sensitive underwater wireless sensor networks. Sci. China Inf. Sci..

[4] Li N., Martínez J.F., Meneses Chaus J.M., Eckert M. (2016). A survey on underwater acoustic sensor network routing protocols. Sensors.

[5] Qian L., Zhang S., Liu M., Zhang Q. A MACA-Based Power Control MAC Protocol for Underwater Wireless Sensor Networks. Proceedings of the IEEE/OES Ocean Acoustics (COA).

[6] Kacimi R., Dhaou R., Beylot A.L. (2013). Load balancing techniques for lifetime maximizing in wireless sensor networks. Ad Hoc Netw..

[7] Darehshoorzadeh A., Boukerche A. (2015). Underwater sensor networks: A new challenge for opportunistic routing protocols. IEEE Commun. Mag..

[8] Han G., Jiang J., Bao N., Wan L., Guizani M. (2015). Routing protocols for underwater wireless sensor networks. IEEE Commun. Mag..

[9] Ahmadi A., Shojafar M., Hajeforosh S.F., Dehghan M., Singhal M. (2014). An efficient routing algorithm to preserve k-coverage in wireless sensor networks. J. Supercomput..

[10] Naranjo P.G.V., Shojafar M., Mostafaei H., Pooranian Z., Baccarelli E. (2016). P-SEP: A prolong stable election routing algorithm for energy-limited heterogeneous fog-supported wireless sensor networks. J. Supercomput..

[11] Shojafar M., Pooranian Z., Naranjo P.G.V., Baccarelli E. (2017). FLAPS: Bandwidth and Delay-Efficient Distributed Data Searching in Fog-Supported P2P Content Delivery Networks. J. Supercomput..

[12] Bai W., Wang H., Shen X., Zhao R., Zhang Y. (2016). Minimum delay multipath routing based on TDMA for underwater acoustic sensor network. Int. J. Distrib. Sens. Netw..

[13] Al Salti F., Alzeidi N., Arafeh B.R. (2016). EMGGR: An energy-efficient multipath grid-based geographic routing protocol for underwater wireless sensor networks. Wirel. Netw..

[14] Ali T., Jung L.T., Faye I. (2014). End-to-end delay and energy efficient routing protocol for underwater wireless sensor networks. Wirel. Pers. Commun..

[15] Pooranian Z., Barati A., Movaghar A. (2011). Queen-bee algorithm for energy efficient clusters in wireless sensor networks. World Acad. Sci. Eng. Technol..

[16] Wei B., Luo Y.M., Jin Z., Wei J., Su Y. ES-VBF: An energy saving routing protocol. Proceedings of the 2012 International Conference on Information Technology and Software Engineering.

[17] Al-Bzoor M., Zhu Y., Liu J., Reda A., Cui J.H., Rajasekaran S. Adaptive power controlled routing for underwater sensor networks. Proceedings of the International Conference on Wireless Algorithms, Systems, and Applications.

[18] Hu T., Fei Y. (2010). QELAR: A machine-learning-based adaptive routing protocol for energy-efficient and lifetime-extended underwater sensor networks. IEEE Trans. Mob. Comput..

[19] Pompili D., Melodia T., Akyildiz I.F. (2010). Distributed routing algorithms for underwater acoustic sensor networks. IEEE Trans. Wirel. Commun..

[20] Hsu C.C., Liu H.H., Gómez J.L.G., Chou C.F. (2015). Delay-sensitive opportunistic routing for underwater sensor networks. IEEE Sens. J..

[21] Nowé A., Brys T. (2016). A Gentle Introduction to Reinforcement Learning. Scalable Uncertainty Management.

[22] Zhang Y., Chen Y., Zhou S., Xu X., Shen X., Wang H. (2016). Dynamic node cooperation in an underwater data collection network. IEEE Sens. J..

[23] Xie P., Cui J.H., Lao L. VBF: Vector-Based Forwarding Protocol for Underwater Sensor Networks. Proceedings of the International Conference on Research in Networking.

[24] Su Y., Zhu Y., Mo H., Cui J.H., Jin Z. UPC-MAC: A Power Control MAC Protocol for Underwater Sensor Networks. Proceedings of the International Conference on Wireless Algorithms, Systems, and Applications.

[25] Yan H., Zhou S., Shi Z.J., Li B. A DSP implementation of OFDM acoustic modem. Proceedings of the Second Workshop on Underwater Networks.

[26] Molins M., Stojanovic M. Slotted FAMA: A MAC protocol for underwater acoustic networks. Proceedings of the IEEE Oceans.

